# Dr. J. Harris on Springing of Plates

**Published:** 1853-01

**Authors:** 


### DR. J. HARRIS ON SPRINGING OF PLATES.

We have a postcript from Dr. Harris in relation to this subject ; but too late to appear with his article ; assigning as another cause of the springing of plates, the arranging of the teeth in contact with each other, and he notices with commendation the proposition to put a piece of paper between the teeth. We have noticed that when the teeth are neatly adjusted together, and the plate springs, that the gum is often scaled off in places. Would this, however, be the case, if the plates had not sprung? The paper burning out would give room; and we have thought more than would look well.

